# Antitrypanosomal and Cytotoxic Activities of 22-Hydroxyclerosterol, a New Sterol from *Allexis cauliflora* (Violaceae)

**DOI:** 10.3797/scipharm.1012-10

**Published:** 2011-02-07

**Authors:** Yves Oscar D. Nganso, Igor Eric W. Ngantchou, Ernestine Nkwenoua, Barthelemy Nyasse, Colette Denier, Véronique Hannert, Bernd Schneider

**Affiliations:** 1 Laboratory of Medicinal Chemistry & Pharmacognosy, Department of Organic Chemistry, Faculty of Sciences, University of Yaoundé I, Box 812 Yaoundé, Cameroon; 2 UMR 5068, LSPCMIB, Université Paul Sabatier, Bât. II R1, 118 Route de Narbonne, 31062 Toulouse, Cedex 4, France; 3 Research Unit for Tropical Diseases, de Duve Institute, TROP 74.39, Avenue Hippocrate 74, B-1200 Brussels, Belgium; 4 Max Planck Institute for Chemical Ecology, Beutenberg Campus, Hans-Knöll-Str. 8, D-07745 Jena, Germany

**Keywords:** Clerosterol, Enzyme inhibitor, Trypanocide, *Trypanosoma brucei*, Stigmastane sterols, Cytotoxicity, NMR, Structure elucidation, Natural products

## Abstract

In the search for new antiparasitic natural compounds from the medicinal plants from Cameroon, the new 22-hydroxyclerosterol, established as such on the basis of detailed chemical and spectroscopic analysis, was isolated from the hexane extract of the stem bark of *Allexis cauliflora* together with the known clerosterol. 22-Hydroxyclerosterol inhibited the growth of *Trypanosoma brucei brucei* cells with an ED_50_ value of 1.56 μM. The compound was also established as an uncompetitive inhibitor of the glycolytic enzyme PGI of *T. brucei* (Ki’= 3 ± 1 μM), an uncompetitive inhibitor of mammalian rabbit muscles’ enzyme PyK (Ki’= 26 ± 3 μM) and a mixed inhibitor of PyK of *Leishmania mexicana* (Ki’= 65 ± 10 μM; Ki= 24 ± 5 μM).

## Introduction

*Allexis cauliflora,* belonging to the family Violaceae, is commonly distributed from Congo to Cameroon [1]. Its stem bark is used in Cameroonian folk medicines to treat fever and syphilis [2]. Despite the availability of this ethnopharmacological information, there is no phytochemical report published on the genus *Allexis* to date. However, previous phytochemical studies on some species of other Violaceae genera indicated the occurrence of potentially useful medicinal secondary metabolites such as flavonoids and triterpenoids. They have been reported as matrix metalloproteinase inhibitors [3], topoisomerase inhibitors [4], antioxidants [5] and antiplasmodials [6]. Following an ethnopharmacological survey on medicinal plants used against parasitic diseases in Cameroon, the species *A. cauliflora* emerged as one of the most commonly used plants in the management of such diseases. As the result of that information, and as a continuation of our efforts to add value to medicinal plants of Cameroon, biological activity and the cytotoxicity profile of *A. cauliflora* were studied in an attempt to scientifically support its ethnomedical applications.

In this report, chemical constituents of the stem bark of this small tree are described and, specifically, analysed as potential inhibitors of both glycolytic enzymes and the growth of *Trypanosoma brucei brucei* bloodstream form (strain 427). The isolated compounds were also tested on MRC-5 mammalian cells (fibroblasts) in order to establish their toxicity profile.

## Results and Discussion

### Chemistry: structural elucidation of isolated compounds

**Fig. 1.**
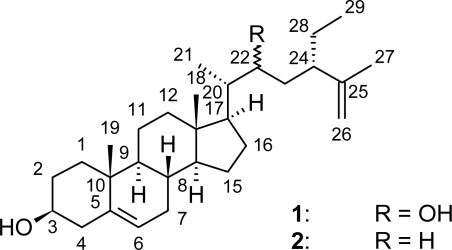
Chemical structures of compounds **1** and **2**

Chromatographic partitioning of the *n*-hexane extract (9.0 g) of dry stem bark (2.9 kg) of *A. cauliflora* on silica gel using *n*-hexane/ethyl acetate mixtures of increasing polarity, led to the isolation of the new 22-hydroxyclerosterol (**1**) and the known clerosterol (**2**). The structures of the two compounds (Figure 1) were established on the basis of detailed chemical and spectroscopic analyses and by comparison with previously reported data on clerosterols [7–10]. The known compound **2** was identified as clerosterol by combined analyses of HREIMS (*m/z* 412 [M^+^], NMR spectroscopic data (^1^H NMR, ^13^C NMR, ^1^H-^1^H COSY, HSQC and HMBC) and by comparing its spectroscopic and physical data with those available in the literature on clerosterol [7, 8].

The new compound **1** (mp: 166–168 °C) gave a positive reaction to the Liebermann-Buchard reagent, suggesting its sterol nature. The ^13^C NMR spectra of compound **1** displayed signals of 29 carbon atoms that were similar to those of compound **2**. A detailed comparison of the ^13^C NMR chemical shift values of the two compounds (Table 1) indicated that they had the same 3β-hydroxy-Δ^5^ sterol ring system and they differed only in the functionality of their side chains. A striking difference between compounds **1** and **2** was observed in the ^13^C chemical shifts for carbon numbered C-22 of the side chain. The signal of this side chain carbon (C-22) was not only dramatically shifted from δ 33.7 in the spectrum of **2** to a lower field (δ 70.7) in the spectrum of **1** but, as indicated by DEPT spectra, also changed from a methylene in compound **2** to a methine in compound **1**. Furthermore, HMBC and HSQC spectra revealed that the proton at C-22 in compound **1** (δ 3.6), attached to a carbon atom bearing an oxygen, belong to the side chain as evidenced by a network of correlations between the carbon C-22 and the carbon atoms at δ 12.5 (C-21), 45.7 (C-24) and 52.9 (C-17) through three bonds and with those at δ 33.1 (C-23) and 42.4 (C-20) through two bonds, respectively. Additionally, the difference between compounds **1** and **2** was obtained from the mass spectra which revealed that the molecular mass of **1** (*m/z* 428 [M^+^]) was greater by 16 units than that of **2,** thus confirming the presence of an extra oxygen atom in compound **1**. Based on these evidences, the structure of compound **1** was established as 22-hydroxyclerosterol, a new derivative of clerosterol (Figure 1). Further heterocorrelation across signals in the ring system and the side chain were fully consistent with the proposed structure. Complete ^1^H and ^13^C NMR data of 22-hydroxyclerosterol (**1**) together with HMBC (^2^*J*_CH_, ^3^*J*_CH_) correlations are presented in Table 1.

### Biological assays

#### In vitro assays of compounds 1 and 2 on Trypanosoma brucei cells

The isolated compounds **1** and **2** were tested on *Trypanosoma brucei brucei* bloodstream form cells cultured *in vitro* as well as on some glycolytic enzymes in order to establish their trypanocidal activity. The results obtained indicated that compound **1**, with an ED_50_ value of 1.56 μM on the growth of *Trypanosoma brucei brucei*, was more active on *T. brucei* cells than previously studied sterols such as saringerol and 24-hydroxyperoxy-24-vinylcholesterol displaying IC_50_ values ranging from 3.2 to 7.8 μM [11]. Compound **1** was 86 fold more potent than compound **2** (ED_50_ 134.34 μM) which is known to exhibit weak antitrypanosomal activity and to be non toxic to mammalian cells [11].

The significant trypanocidal activity of compound **1** in comparison with that of compound **2** (Table 2) raised a question on its toxicity profile. In this context and for a selectivity concern, compound **1** was tested on growth of the mammalian fibroblast cell line. The result obtained indicated that compound **1** inhibited both mammalian cells and growth of the *T. brucei* cells at quite similar concentrations (ED_50_ 1.12 μM and ED_50_ 1.56 μM, respectively). This cytotoxicity could be explained by the presence of the hydroxyl group at C-22 in its side chain since compound **2**, lacking the hydroxyl functionality in its side chain, is non toxic on mammalian cells.

#### Inhibition of three glycolytic enzymes of T. brucei by isolated compounds

Following the significant activity (ED_50_= 1.56 μM) of compound **1** on the growth of *T. brucei* cells, and in an attempt to establish its mechanism of action, it was deemed necessary to test isolated compounds as potential inhibitors of some glycolytic enzymes (Aldolase, PGK, PFK, PGI, GAPDH and PyK).

A global analysis of obtained results (Table 3) showed that compound **1** was selective of the parasite enzyme. It inhibited GAPDH and PGI of *T. brucei* with an IC_50_ value of 30 μM.

Meanwhile, compound **2** was only active on *T. brucei* PGI and selective of the enzyme with an IC_50_ value of 45 μM. It has also been shown that **1** was more active on rabbit muscle pyruvate kinase (PyK) than on its isolated homologue from *Leishmania mexicana*.

Kinetic studies showed that compound **1** was an uncompetitive inhibitor of the phospoglycerate isomerase (PGI) enzyme. Compound **1** inhibited the enzyme (Ki’= 3 ± 1 μM) by binding the fructose-6-phosphate (F6P)/PGI complex with an affinity 76-fold more important than that of the natural substrate (F6P) to the enzyme (PGI). Moreover, it was also shown to be an uncompetitive inhibitor of PyK of rabbit muscle (Ki’= 26 ± 3 μM) and a mixed inhibitor of Pyk of *Leishmania mexicana* (Ki’= 65 ± 10 μM; Ki= 24 ± 5 μM); whereas compound **2** was successfully shown to be a mixed inhibitor of PGI of *T. brucei* (Ki= 10 ± 2 μM; Ki’= 68 ± 5 μM).

In conclusion, the isolation and structural elucidation of clerosterol (**2**) and its new isomer 22-hydroxyclerosterol (**1**) from stem bark of *Allexis cauliflora* have been achieved using chemical and spectroscopic methods. Compound **1** has been shown to be more toxic than compound **2** on *T. brucei* cells. Obtained results for clerosterol are in agreement with those observed by Hoet et al. in 2007 [12].

## Experimental

### General experimental procedures

Melting points were determined on a Büchi Melting point apparatus B-540 and are uncorrected. NMR spectra (^1^H and ^13^C) were recorded using a Bruker DRX 500 spectrometer in CDCl_3_, with TMS as internal standard at room temperature. Chemical shifts are given in δ and coupling constants (*J*) in Hz. The EIMS spectra were obtained at 70 eV in a VG Autospec apparatus. HRMS spectra were recorded on a MasSpec sector field mass spectrometer (Micromass Ltd., Manchester, UK) with a direct insertion probe Column chromatography was performed using silica gel (0.063–0.2 mm/70–230 mesh) from Merck. Visualization of the compounds on the chromatographic plates was made under ultraviolet light, exposure to iodine vapor or spraying with 50% diluted sulphuric acid in water, followed by gentle heating.

### Plant material

The stem bark of *A. cauliflora* (Violaceae) were collected at Ebolowa on the Elephants Hill, South Province of Cameroon, in July 2007, and identified by Mr. Nana of the Cameroonian National Herbarium (Yaoundé) where a Voucher specimen (HNC 18374) has been deposited.

### Extraction and isolation

Air-dried pieces (2.9 kg) of stem bark of *A. cauliflora* were powdered and macerated with *n*-hexane (10 l, 72 h) at room temperature. The residue obtained upon evaporation of solvent to dryness afforded 9 g (0.31%) of crude *n*-hexane extract.

The major part of the *n*-hexane extract (8.5 g) was then fractionated over an open silica gel column using *n*-hexane/EtOAc mixtures of increasing polarities as eluents. The *n*-hexane/EtOAc (19:1) fraction afforded 104.8 mg (1.23%) of the known clerosterol (**2**). Further elution with increasing EtOAc concentration afforded 241.4 mg (2.84%) of the new 22-hydroxyclerosterol (**1**) at *n*-hexane/EtOAc (9:1). The chemical structures (Figure 1) of isolated compounds were determined by means of spectroscopic methods (^1^H-NMR, ^13^C-NMR, ^1^H-^1^H COSY, HSQC and HMBC) and by comparing spectroscopic and physical data with those of the literature [7–10].

#### (3β,24S)-Stigmasta-5,25-diene-3,22-diol (22-Hydroxyclerosterol, **1**)

White powder, M.p.: 166–168°C. ^1^H-NMR (500 MH_Z,_ CDCl_3_): δ 0.69 (3H, s H-18), 0.83 (3H, t, *J* = 7.3Hz, H-29), 0.93 (3H, d, *J* = 6.8Hz, H-21), 0.93 (1H, H-9) 0.97 (1H, H-14), 1.01 (3H, s, H-19), 1.07 (1H, H-1b), 1.07 (1H, H-17), 1.09 (1H, H-15b), 1.17 (1H, H-12b), 1.28 (1H, H-23a), 1.30 (1H, H-16b), 1.33 (1H, H-23b), 1.36 (2H, H-28), 1.46 (1H, H-11b), 1.5 (1H, H-2b), 1.5 (1H, H-7b), 1.5 (1H, H-8), 1.50 (1H, H-11a), 1.58 (1H, H-15a), 1.61 (3H, s, H-27), 1.65 (1H, H-16a), 1.66 (1H, H-20), 1.84 (1H, H-2a), 1.85 (1H, H-1a), 1.97 (1H, H-7a), 2.01 (1H, H-12a), 2.21 (1H, H-24), 2.23 (1H, ddd, *J* = 12.9, 5.2, 2.1 Hz, H-4b), 2.29 (1H, H-4a), 3.52 (1H, m, H-34), 3.62 (1H, ddd, *J* = 9.8, 3.2, 2.8 Hz, H-22), 4.77 (1H, H-26a), 4.83 (1H, H-26b), 5.35 (1H, dis. t, *J* = 5.2 Hz, H-6). HREIMS: *m/z* 428.366308 [M]^+^, calcd for C_29_H_48_O_2_: 428.365431.

#### (3β,24S)-Stigmasta-5,25-dien-3-ol (Clerosterol, **2**)

White powder, M.p.: 120–122°C (lit. [7] 120–121°C). ^1^H NMR (500 MHz, CDCl_3_ selected signals): δ 0.67 (3H, s H-18), t, *J* = 7.3 Hz, H-29), 0.91 (3H, d, *J* = 6.8 Hz, H-21), 1.01 (3H, s, H-19), 1.56 (3H, s, H-27), 3.52 (1H, m, H-3), 4.64, 1H, H-26a), 4.73 (1H, H-26b), 5.35 (1H, H-6). For ^13^C NMR, see Table 1. HREIMS: *m/z* 412.368935 [M]^+^, calculated for C_29_H_48_O: 412.370517.

### Biology essay procedure

#### Cell culture

*Trypanosoma brucei brucei* (strain 427) bloodstream forms were cultured in HMI-9 medium containing 10% heat-inactivated [12]. The mammalian fibroblast cell line MRC-5 was grown in DMEM (Gibco) supplemented with 10% heat-inactivated FBS and extra glutamine. Cells were grown at 37 °C in humidified incubators in an atmosphere of 5% CO_2_ [13].

#### Antitrypanosomal and cytotoxicity assays

The assay performed, the long incubation low inoculation test (LITIT), was previously described by Räz et al., 1997 [14] with minor modifications by Baltz et al., 1985 [15]. To evaluate the selectivity of the *in vitro* antitrypanosomal activity, *in vitro* cytotoxicity tests were also performed. The same general method was used in order to facilitate the comparison.

The compounds were initially dissolved in DMSO at a concentration of 20 mg/ml and diluted with the medium. Series of dilution were done in 96-well plates; each dilution was tested in duplicate. Cells in suspension were thereafter added to each well at a density such as, after 72 h of incubation, in control wells the culture reached the end of logarithmic growth phase (*T. brucei*), or adhesive cells have formed a confluent monocellular film (mammal cells MRC-5). The highest concentration of DMSO was 0.5%. After 72 h of incubation, Alamar Blue, a marker of cell viability, was added in each well and fluorescence was quantified after a total incubation time of 76 h at an excitation wavelength of 530 nm and at an emission wavelength of 590 nm. ED_50_ values were calculated by linear interpolation. Assays with commercial drugs were also carried out in order to have reference values. All results represent average values obtained in at least three independent experiments, each experiment comprising a duplicate set of tests.

## Figures and Tables

**Tab. 1.** ^13^C NMR Chemical Shifts of compounds **1** and **2** (CDCl_3_, 125 MHz, TMS).

**#**	**Compound 1**		**Compound 2**

	**δc**	**HMBC of attached H**	**δc**
1	37.3		37.3
2	31.7		31.7
3	71.8	C-1, C-2, C-4	71.8
4	42.3		42.3
5	140.8		140.8
6	121.6	C-4, C-7, C-10	121.7
7	31.9		31.9
8	31.9		31.9
9	50.2		51.1
10	36.5		36.5
11	21.1		21.1
12	39.8		39.8
13	42.7		42.3
14	56.4		56.8
15	24.4		28.2
16	27.5		29.4
17	52.9		56.1
18	11.8	C-12, C-13, C-14, C-17	11.8
19	19.4	C-1, C-5, C-10, C-11	19.4
20	42.4		35.5
21	12.5	C-17, C-20, C-22	18.7
22	70.7	C-17, C-20, C-21, C-23, C-24	33.7
23	33.1		24.3
24	45.7		49.5
25	147.0		147.6
26	112.6	C-24, C-27	111.4
27	17.7	C-24, C-25, C-26	17.8
28	27.0		26.5
29	12.1	C-24, C-28	12.1

**Tab. 2.** Cytotoxicity of isolated compounds (ED_50_, μM)

**Compound**	***Trypanosoma brucei* (strain 427)**	**Mammalian cells (MRC-5 fibroblasts)**
**1**	1.56	1.12
**2**	134.34	no effect

**Tab. 3.** Enzymatic inhibition (IC_50_, μM)

	**PGI**		**GAPDH**		**PyK**	

**Compound**	***T. brucei***	**Rabbit muscle**	***T. brucei***	**Rabbit muscle**	***L. mexicana***	**Rabbit muscle**
**1**	30 ± 10	n.i.	30 ± 10	n.i.	30 ± 5	80 ± 10
**2**	45 ± 10	n.i.	n.i.	n.i.	n.i.	n.i.

n.i. … no inhibition observed at a concentration of 50 μM.
